# The effect of statins therapy in aortic stenosis: Meta-analysis comparison data of RCTs and observationals

**DOI:** 10.1016/j.dib.2016.02.045

**Published:** 2016-02-26

**Authors:** Ying Zhao, Rachel Nicoll, Yi hua He, Michael Y. Henein

**Affiliations:** a Ultrasound Department, Beijing Anzhen Hospital, Capital Medical University, Beijing 100029, China; b Department of Public Health and Clinical Medicine, Umeå University, Umeå, Sweden

## Abstract

Aortic stenosis has been shown to share the same risk factors as atherosclerosis which suggested a potential benefit from statins therapy. Fourteen studies which provided the effect of statins treatment on aortic stenosis (AS) were meta-analyzed, including 5 randomized controlled trials (RCTs) and 9 observational studies. In the RCTs, statins did not have any influence on peak aortic valve velocity, peak valve gradient, mean valve gradient, aortic valve area and aortic calcification compared to controls. In the observational studies, the peak valve velocity, peak gradient and aortic valve area showed less progression in the statins group compared to controls. This article describes data related article title “The effect of statins on valve function and calcification in aortic stenosis: a meta-analysis” (Zhao et al., 2016) [1].

**Specifications table**

**Table:** 

Subject area	*Clinical research, Meta-analysis*
More specific subject area	*Medicine*
Type of data	*Figure*
How data was acquired	*Meta-analysis*
Data format	*Analyzed*
Experimental factors	*Aortic stenosis parameters*
Experimental features	*14 eligible studies selected from Pubmed*
Data source location	*Canada, Austria, Norway, Portugal, Spain, USA, UK, Italy, Germany, Iran*
Data accessibility	*Data is with this article*

**Value of the data**

• These data segregate the results of the randomized controlled trials (RCT) and the observational studies that assessed the use of statins in patients with aortic stenosis.
• The data show clearly that the RCTs refuted any potential benefit of statins in aortic stenosis in contrast to the observational studies which showed potential slow progression, although not consist.
• This clear discrimination should assist and guide future trials to better accurate ways for assessing the effect of statins in aortic stenosis.
• Researchers might also be allowed in including our data analysis as part of future trials or meta-analyses.

## 1 Data

**Fig. 1:**
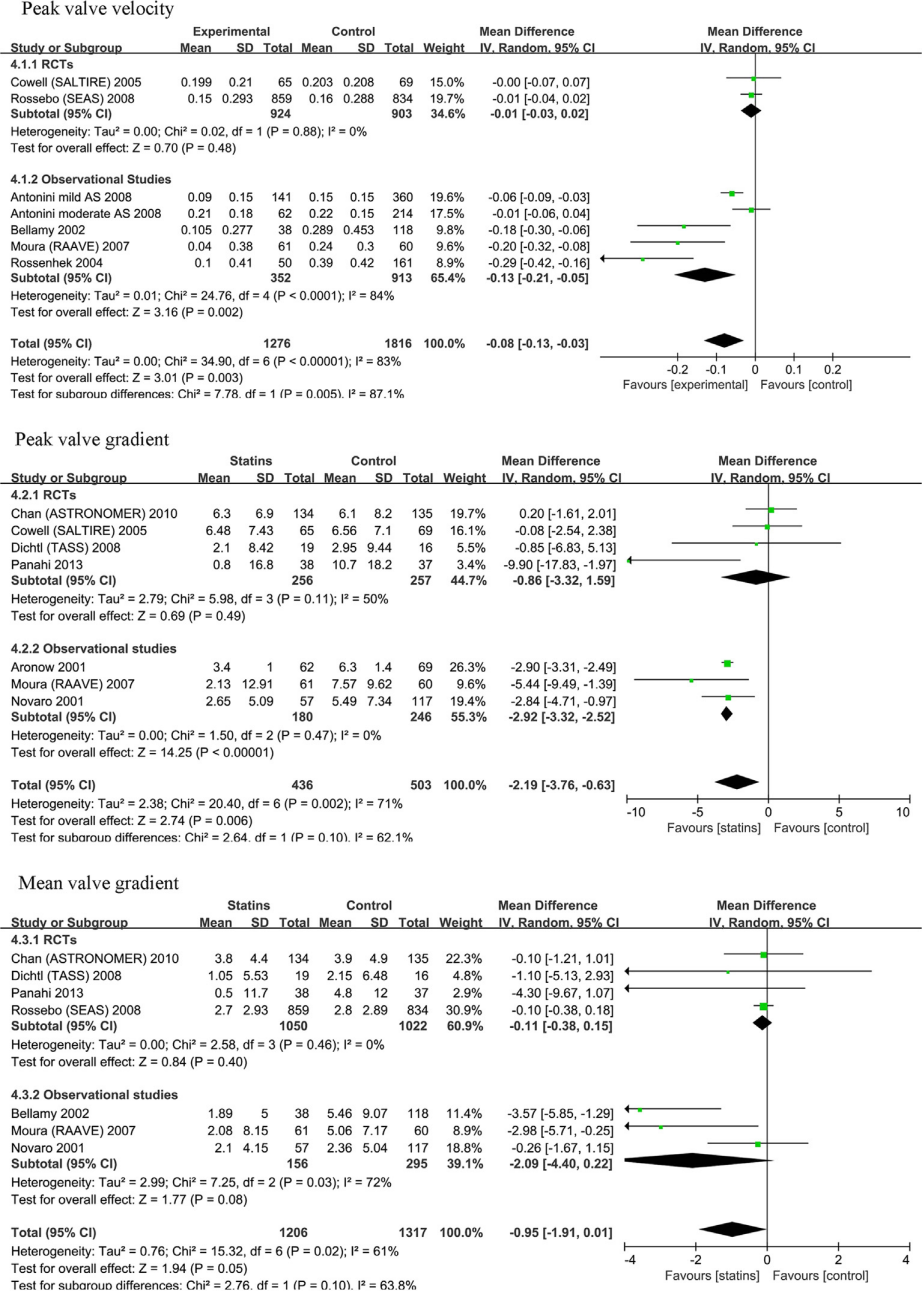
The statins group had less increase in annual peak valve velocity (*p*=0.003) and peak gradient (*p*=0.006) in total, and this was only in observational studies but not in RCTs. The annual mean gradient did not show any significant change in total (*p*=0.05), observationals and RCTs subgroups.

**Fig. 2:**
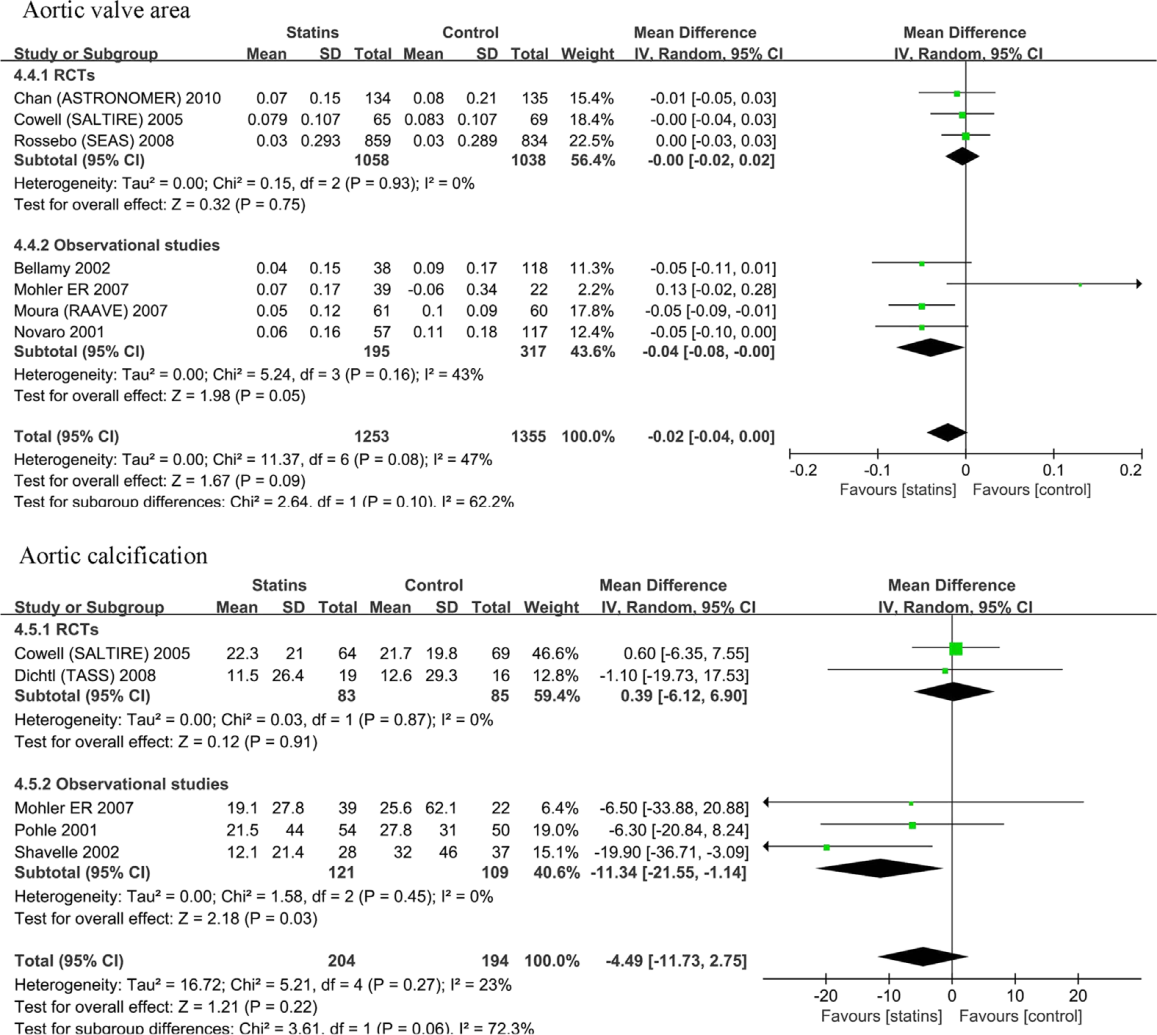
There was no difference between statins and non-statins treatment regarding the annual changes of aortic valve area (*p*=0.09),also it was the case between observational and RCTs subgroups. The annual increase of aortic valve calcification did not show any significant changes between statins and control groups (*p*=0.22) and in RCTs subgroups, but it was not the case in observational subgroups.

The data presented here showed the statins group (as a whole) had less increase in annual peak valve velocity (*p*=0.003), annual peak gradient (*p*=0.006)). All these were only observed in observational studies (*p*=0.002, *p*<0.001) but not in RCTs (*p*=0.48, *p*=0.49). The annual mean gradient showed no any significant changes between statin and control groups (*p*=0.05), and it was the case in observational subgroup (*p*=0.08) and in RCTs subgroup (*p*=0.40) (Fig. 1). While, the statins group had a trend of less changes in aortic valve area (*p*=0.05) and less changes in aortic calcification (*p*=0.03) in observational subgroup but not in RCTs subgroup (*p*=0.75 and *p*=0,91) and in total (*p*=0.09 and *p*=0.22) (Fig. 2).

## 2 Experimental design, materials and methods

### 2.1 Design, materials and methods

We searched a medical database (PubMed) using the MeSH keywords (“aortic valve stenosis” and “Hydroxymethylglutaryl-CoA Reductase Inhibitors”) together and in combination, having limited the search to studies reported only in English prior to April 2015 and those which used adults ≥19 years of age. Fourteen studies were identified including 5 RCTs [2], [3], [4], [5], [6] and 9 observational studies [7], [8], [9], [10], [11], [12], [13], [14], [15]. The mean follow-up period of the studies ranged between 12 months and 5.6 years. Data from patients commenced on statins were compared with controls and between subgroups.

### 2.2 Statistical analysis

The data was extracted from each study and analyzed using the Revman software 5.3. The annual changes in peak aortic velocity, peak and mean valve gradient, aortic valve area and aortic calcification were compared between the statins group and the control group as well as the subgroups. A *p*<0.05 was defined as statistical significance.
